# Validation of the Novel Interprofessional Shared Decision-Making Questionnaire to Facilitate Multidisciplinary Team Building in Patient-Centered Care

**DOI:** 10.3390/ijerph192215349

**Published:** 2022-11-21

**Authors:** Yuko Goto, Hisayuki Miura

**Affiliations:** Department of Home Care and Regional Liaison Promotion, National Center for Geriatrics and Gerontology, Obu 474-8511, Aichi, Japan

**Keywords:** shared decision-making, multidisciplinary team care, educational tool, advance care planning

## Abstract

To support patients in making complex and difficult decisions, it is necessary to form a team that comprises different specialists, the patient, and family members who have detailed information about the latter. Shared decision-making (SDM) is the foundation of patient-centered care; however, its structure in the context of multidisciplinary teams remains unclear. This cross-sectional study aimed to validate the novel interprofessional SDM measure (“Group’s SDM measure”). We used data of 175 participants who attended SDM Workshops for Advance Care Planning. The Group’s SDM measure included 10 Japanese items that could be used by small groups of multidisciplinary professionals, and the responses were rated using a 6-point Likert scale. The index exhibited a single-factor structure and high goodness of fit with residual correlation via factor analysis. We calculated Cronbach’s α (α = 0.948) and McDonald’s ω (ω = 0.948) and verified high internal consistency. The Group’s SDM measure can be used when evaluating the SDM process where multidisciplinary professionals are involved. We hope that in the future, it will lead to the promotion of interprofessional SDM through training with the use of this measure.

## 1. Introduction

In Japan, the number of older patients continues to increase, and there are many patients who need support in making complex and difficult decisions (not only for treatment but also for nursing and home health care). To support patients in making difficult decisions in a clinical practice, the involvement of multidisciplinary professionals with different specialties and the exchange of knowledge are both important. Multiple guidelines [1,2] recommend multidisciplinary team decision support when making important decisions, especially for older people with chronic illnesses. Multidisciplinary teams are organized in a variety of settings, including within a hospital, within a community, and between a hospital and a community. At these settings, medical, nursing care, and welfare professionals often conduct conferences together, such as at pre-discharge conferences and regular conferences in home care.

The acquisition of shared decision-making (SDM) skills is important when making complex decisions, including cases of clinical uncertainty [3,4]. It has been demonstrated that encouraging communication between the patient and the professional during SDM promotes mutual understanding [5], reduces conflicts and disagreements arising during the decision-making process, increases patient satisfaction [6], improves patient adherence, and prolongs treatment effects [7,8].

Previous reports proposed the use of SDM by a team [9], and it has been proven that multidisciplinary professionals’ provision of ongoing support for decision-making regarding palliative, transitional, and primary care on a routine basis as a team enhances patient participation [6,10,11]. Especially for patients who have had strokes who need continuous decision-making support over an extended period—from the acute phase to the convalescence stage and during the home healthcare stage—there is a need for treatment as well as support by an interdisciplinary team, which includes a home healthcare team, daily care team, and nursing professionals [12,13,14]. In Japan, where the elderly population is increasing, the promotion of SDM by a team that supports various types of difficult decision-making processes is required.

Measures of SDM skills have been developed globally [15,16,17], including in Japan [18,19,20]. Many of these measures are indicators for medical decisions between patients and physicians [15,16,17,18,19] or between patients and healthcare professionals other than physicians [20]; however, even globally, there are few indicators [21] that assume multidisciplinary team collaboration scenes, such as joint conferences. At present, there are no recognized evaluation indices in Japan that can be used by teams for practical SDM and associated training.

The authors developed an advance care planning training program incorporating SDM skills training and evaluated its usefulness [22]. In the workshops of the training program, measures of SDM skills [18,19,20] were used in role-playing sessions, and it was confirmed that the participants’ SDM skills improved. However, these measures were for one-to-one conversation between a patient and a healthcare professional. For this training program, the authors developed the novel interprofessional SDM questionnaire to facilitate multidisciplinary team building in patient-centered care and used it to measure the quality of group discussion by multidisciplinary professionals. However, validation of the novel measure has never been to done to date.

Therefore, in this study, we aimed to validate the novel interprofessional SDM questionnaires to be used for practical or educational purposes.

## 2. Materials and Methods

### 2.1. Design

This study followed a cross-sectional design. To confirm the construct validity, concurrent validity, and reliability of the preliminary version of our evaluation index (Group’s SDM measure), we conducted a quantitative analysis.

### 2.2. Measurements

#### 2.2.1. Identification of Elements Needed for the Interprofessional SDM Measure (Group’s SDM)

**Figure 1:**
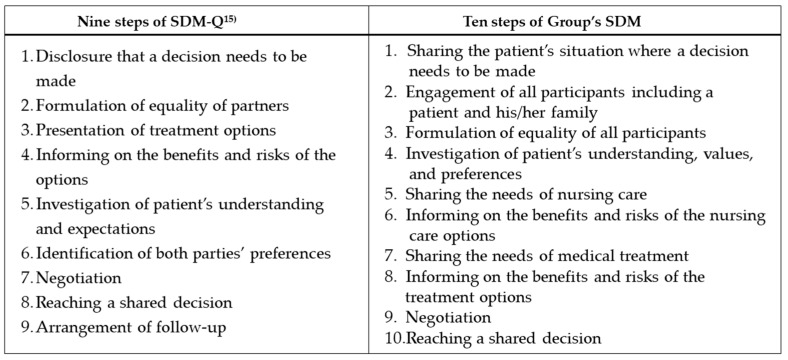
Steps in the SDM-Q process and Group’s SDM.

Although various definitions of SDM are currently being discussed, the idea of SDM is characterized by several key features: (1) the participation of at least two persons, that is, the patient and the professional; (2) both parties make decisions through sharing information about treatment options, patients’ values, and preferences [23]. The definition of SDM was further clarified by eliciting points of view discussed in the previous literature [23]. Based on these reports, Simon et al. defined the sequential steps of the SDM process (nine steps of SDM-Q presented in Figure 1), and Kriston et al. developed the nine-item questionnaire (SDM-Q-9) based on Simons et al.’s study [15]. The questionnaire has been translated into over 30 languages and is used worldwide [24]. The authors validated the Japanese version [18].

In this study, the authors (YG, HM) identified the elements needed to assess interprofessional SDM theoretically based on SDM-Q. As background for creating this measure, a team of multidisciplinary professionals was required to support a complex and difficult decision-making process [12,13,14]. The authors designed the interprofessional SDM measure based on the assumption that multidisciplinary professionals will understand that SDM is carried out by a small team comprising a patient and professionals and that professionals who have learned the skills required for such SDM will be involved in providing support to patients and their families in making decisions during complex and difficult situations.

In Japan, it is crucial to communicate goals, identify problems, and reach consensus through conversation to enable professionals with different specializations, such as medical and nursing care, to work together as a team [25]. Therefore, the authors integrated the issues related to multiple Japanese professional collaboration and team formations with the SDM concept; this concept was then considered by the research group while developing the Group’s SDM measure.

As a result, the authors identified 10 elements of interprofessional SDM (10 steps of the Group’s SDM presented in Figure 1) based on SDM-Q. A measure consisting of 10 elements, assuming the participation of patients and their families, was designed. Specifically, the authors included items about treatment and care options (items 5 and 7), items for comparing and considering these options (items 6 and 8), and items about criteria for values used to select an option (items 4 and 9). We also included items for understanding important information about team formation (items 1–3), building equal relationships (item 3), considering directions (item 9), and building consensus (item 10). Item 9 of the SDM-Q, “Arrangement of follow-up,” was not included in the novel measure because the need for follow-ups is different between professionals.

#### 2.2.2. Interprofessional SDM Measure (Group’s SDM)

**Figure 2:**
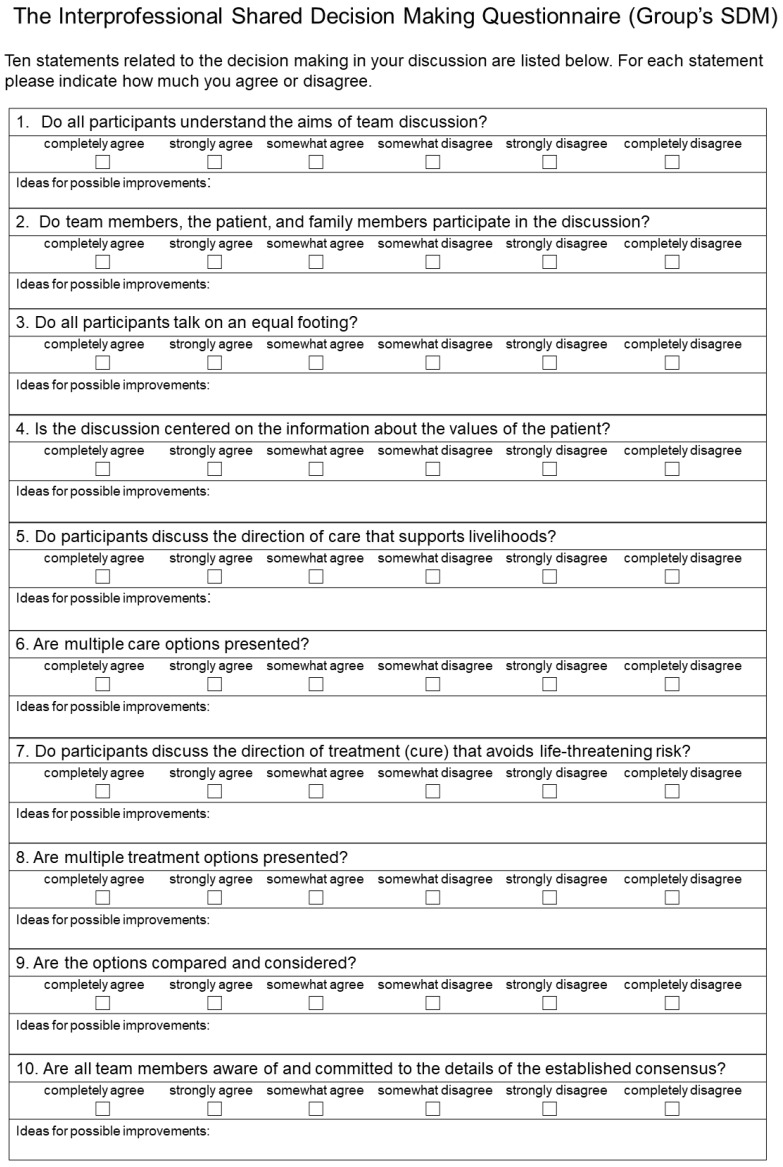
Reverse translation of the Group’s SDM into English.

Based on the elements needed to assess the interprofessional SDM (Figure 1), the authors (YG, HM) developed a 10-item questionnaire (Figure 2). Each question presented in Figure 2 corresponds to each element presented in Figure 1. Responses were provided using a 6-point Likert scale, ranging from “completely disagree” to “completely agree,” to avoid a ceiling effect [15] in the same way as the SDM-Q-9 questionnaires [15,16,18,19].

To stimulate group discussion, the authors added a free-text line about “possible improvement” after each question. The face validity of the Group’s SDM was tested with the help of several healthcare providers.

### 2.3. Participants and Ethical Considerations

We used data from participants in the SDM workshops for Advance care planning (ACP) hosted by seven training centers from June 2020 to October 2022. The participants were medical, nursing, or social care specialists who were clinically involved in providing decision-making support to patients and were potential ACP leaders in their local communities. Each training center recruited participants for their respective workshops.

The three workshops at all seven sites were attended by physicians who support decision-making regarding medical treatment as well as healthcare professionals other than physicians who support decision-making related to care. The seven institutions collected assessment index data from workshop participants and shared it with the researchers managing the project. In this study, we used the SDM evaluation data submitted by the seven workshop sites.

The SDM measure (Group’s SDM) questionnaire used in the group discussion during the second workshop was filled by the participants before the second workshop, i.e., the data obtained after sharing the measure with the participants were used for discussion during the workshop. The present study was conducted after rigorous conflict-of-interest screening and research ethics review by the National Center for Geriatrics and Gerontology.

IRB Approval Code and Name of the Institution: The National Center for Geriatrics and Gerontology approved the study (Approval code: no. 1585 [5 April 2022]).

### 2.4. Setting and Data Collections

“Training on SDM competency in advance care planning” is a program that was conducted during the SDM workshops for ACP, and it included three workshops conducted over a period of 6 months [22]. In the first and third workshops, the participants were trained in one-to-one conversation using the related SDM questionnaires [18,19,20]. In the second workshop, a group discussion was conducted, and data about the responses to the Group’s SDM questionnaire was collected.

The period between the first and second workshops was approximately one month, and the same participants attended both the workshops.

Before the second workshop, the participants viewed the footage of a group discussion and entered their Group’s SDM assessment into the Group’s SDM measure form; for the footage of this group discussion, a model discussion was used. This setting was a multidisciplinary conference in the home of a patient with dementia and dysphagia. Multidisciplinary professionals, such as a physician, visiting nurse, care manager, and visiting paid caregiver, gathered to discuss future treatment and care policies with the patient and their family. For this conference, the authors scripted conversations in which the physician’s paternalism was prominent.

During the second workshop, a group of approximately six participants formed a team based on the evaluation index that each had filled in and discussed SDM and possible improvements.

After the second workshop, the host collected anonymous data on the results of the questionnaires in an electronic format entered by each participant, deleted the identifying information, and provided the data to the researchers.

Due to the exploratory nature of this study, we used a sample size of ≥100 participants, as suggested by COSMIN [26].

### 2.5. Statistical Analyses

The participants’ characteristics were classified based on the responses to the questionnaire filled during the second workshop.

Regarding the scores obtained using the answers to the Group’s SDM measure questionnaire, “completely agree” was rated as 0 and “completely disagree” was rated as 5. The scores obtained out of a total of 50 were multiplied by 2 to obtain the scores out of 100, and the descriptive statistics of these scores was then compiled.

To measure the adequacy of the responses obtained using the Group’s SDM measure, we used the Kaiser–Meyer–Olkin (KMO) test. Then, we used Bartlett’s test of sphericity to verify the correlation between the variables. As the KMO values of approximately 1 indicate more structural simplicity, we considered the sampling to be adequate when the KMO value was >0.5 [27]. The significance level for Bartlett’s test of sphericity was set at *p* ≤ 0.05.

Subsequently, for the exploratory factor analysis (EFA), we verified the number and structure of factors using the principal axis method. Moreover, for the confirmatory factor analysis, based on the number and structure of the estimated factor, we verified the conceptual structure using structural equation modeling (SEM). The SEM’s goodness of fit was considered high, with an χ^2^/df ratio of ≤2, χ^2^ significance level of *p* > 0.05, goodness of fit index (GFI) of ≥0.9, adjusted goodness of fit index (AGFI) of ≥0.9, root mean square error of approximation (RMSEA) of ≤0.05, and comparative fit index (CFI) of ≥0.95.

To evaluate the internal consistency of the Group’s SDM measure data, Cronbach’s α coefficient and McDonald’s ω coefficient were calculated and the data were deemed to be internally consistent if these coefficients were ≥0.8.

IBM SPSS Statistics 29 and IBM SPSS Amos Graphics 29 (IBM Corp., Armonk, New York, NY, USA) were used to analyze the statistics.

## 3. Results

### 3.1. Subsection

The first workshop included 180 participants, and the second included 175 participants.

After the second workshop, 171 questionnaires were submitted, and the recovery rate was 98%. Based on the answer sheets, we observed that the majority (51%) of the participants in the second workshop were nurses, followed by physicians, medical social workers (MSW), and care managers (Table 1). In addition, the majority of the participants (22%) had ≥25 years of clinical experience as a specialist (Table 2).

### 3.2. Exploratory Factor Analysis

After the second workshop, data from 177 sheets of the Group’s SDM measure questionnaire were submitted. This included data from participants who had left the second workshop in the middle of the workshop. The items of the Group’s SDM measure questionnaire were named from Group’s SDM 1 to Group’s SDM 10, and the results were summarized in descriptive statistics (Table 3).

**Figure 3:**
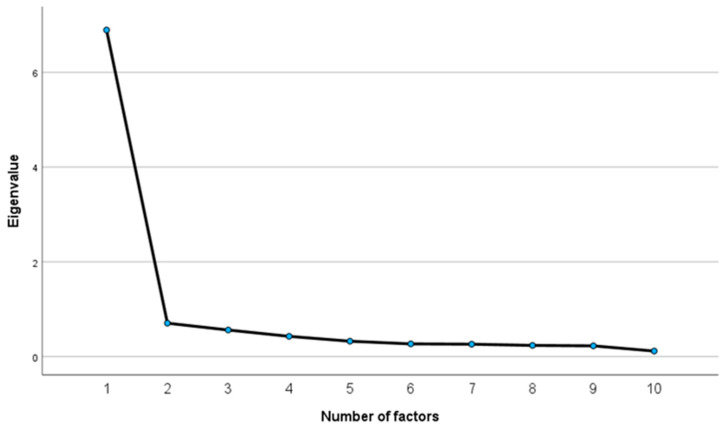
Scree plot of the Group’s SDM measure scores.

The Group’s SDM measure data were analyzed using factor analysis (primary axis method; promax rotation). The resulting KMO value was found to be 0.935, which indicated the appropriateness of the sample size. As Bartlett’s test of sphericity yielded an approximate χ^2^ value of 1510.044 (degree of freedom: 45, *p* < 0.001), we considered that a correlation exists between the items. A scree plot yielded one factor, suggesting a single-factor structure (Figure 3).

Table 4 presents the results of the EFA for the Group’s SDM. The analysis yielded one factor explaining 68.94% of the variance and 6.90 eigenvalue for the entire set of variables. All variables indicated more than 0.4 factor loading and 0.2 communality.

### 3.3. Confirmatory Factor Analysis

The Group’s SDM measure was considered to have a single-factor structure, and this factor was named “Group’s SDM.”

**Figure 4:**
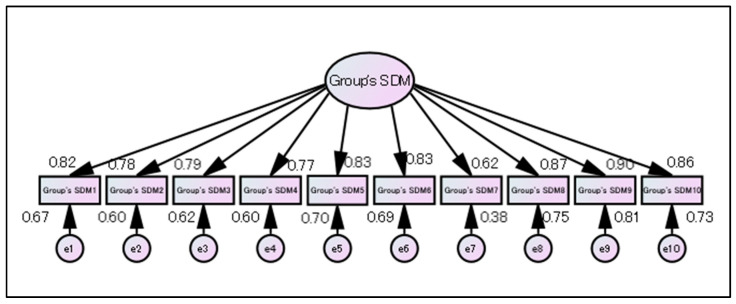
Structural model for the Group’s SDM measure without residual correlation.

When we verified a 10-item single-factor structure with no residual correlation model using SEM, the goodness of fit was found to be poor (χ^2^ ratio = 135.12, χ^2^/df = 1.003, GFI = 0.865, AGFI = 0.788, RMSEA = 0.127, and CFI = 0.933) (Figure 4).

**Figure 5:**
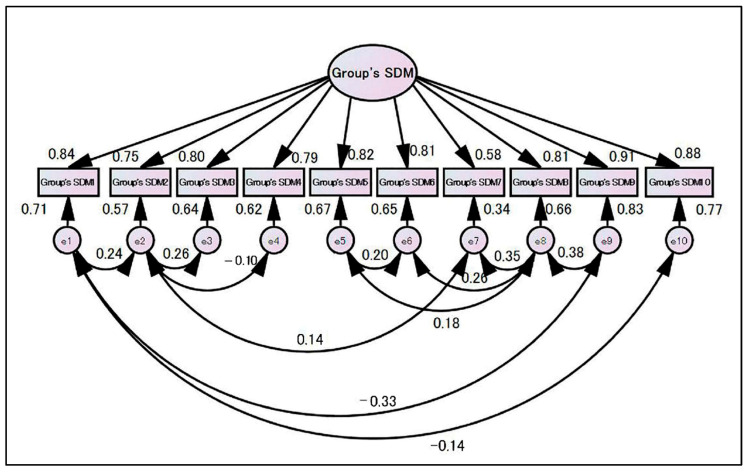
Structural model for the Group’s SDM measure with residual correlation.

Therefore, when we examined the goodness of fit with a model that assumed residual correlation, a model with high fit was completed (χ^2^ ratio = 34.524, χ^2^/df = 1.438, GFI = 0.964, AGFI = 0.917, RMSEA = 0.05, CFI = 0.993) (Figure 5).

### 3.4. Reliability Analysis

To confirm internal consistency, we calculated Cronbach’s α and McDonald’s ω coefficients of the Group’s SDM measure score. The data were confirmed to demonstrate high internal consistency, with α = 0.948 and ω = 0.948.

## 4. Discussion

This study aimed to validate the novel interprofessional SDM measure (Group’s SDM) to be used in practice and education. By analyzing data using this measure, we could confirm its reliability (internal consistency) and validity, which showed the validation for its use in a practical and a training setting.

The distinction between the group and individual decision-making process was elucidated by a previous study [28]. It was suggested that the decision-making process of a small number of professionals and the patient differs from the decision-making process of a team of multiple members with different values and areas of expertise. Therefore, clarification of a team SDM structure is important to advance team SDM practice and education in the future.

### 4.1. Group’s SDM Measure Structure

As interprofessional SDM is conducted by members with diverse values, including multiple professionals, patients, and patients’ families, conflicts of interest within the team may possibly arise. Teams must communicate their support goals, choose the right team members, and exchange their opinions and ideas to provide effective support [29].

The following items in the Group’s SDM measure could be essential for exercising SDM in a team: Team SDM 1 (Do all participants understand the aims of the team discussion?), Group’s SDM 3 (Does the discussion take place with an equal contribution from all members?), Group’s SDM 5 (Does the course of care support the lifestyle of the patient under discussion?), Group’s SDM 7 (Is the course of treatment (cure) aimed at avoiding life-threatening situations discussed?), and Group’s SDM 10 (Are all team members aware of and committed to the details of the established consensus?).

In addition, information from the patient’s perspective and thorough patient participation are expected to increase team transparency and strengthen partnership [30]; Group’s SDM 2 (Do team members, the patient, and family members participate in the discussion?) and Group’s SDM 4 (Is the discussion centered on the information about the values of the patient?) are important items for an effective team SDM.

Furthermore, as SDM is a decision-making support method developed based on the decision theory [31], prospective theory [32], and fuzzy logic [33], Group’s SDM 4 (Is the discussion centered on the information about the values of the patient?), Group’s SDM 6 (Are multiple care options presented?), Group’s SDM 8 (Are multiple care options presented?), and Group’s SDM 9 (Are the options compared and considered?) are important. To support decisions based on patient preferences, it is crucial to understand correct information about each option and consider uncertainty.

Some training programs for interprofessional SDM have been developed [21,34]. Körner [21] et al. employed two independent measures for external (SDM) and internal (interprofessional) participation to evaluate interprofessional SDM in the training program. In this study, they used SDM-Q-9 [15] directly as a measure of external participation; however, this measure was originally developed on the basis of a one-to-one dialog between a patient and a physician, not for the evaluation of a discussion with a large number of people. The measure (Group’s SDM) validated in this study can overcome this issue.

In our previous report, the training program integrating ACP and SDM was found to improve SDM skills through the use of SDM-Q series and confidence of ACP practice [22]. In the study, the effect of the interprofessional SDM (Group’s SDM) was not assessed, but through group discussions using this measure, there is a possibility that patient-centered multidisciplinary collaboration can progress. To evaluate the outcomes, further study is required.

All team members must have communication, leadership, and management skills to be able to effectively support the team given their diverse specialties and values [35]. It is believed that the reason why the Group’s SDM measure did not include these items is that team members supporting complex and difficult decision-making need to involve specialists with knowledge and experience in supporting decision-making with relatively little conflict. Furthermore, it was pointed out that decision-making support requires not only skills and knowledge but also a relationship of trust with the patient and the patient’s family in addition to emotional support [36]. Professionals involved in decision-making support are expected to acquire the skills needed to build trustworthy relationships with patients and their families and provide emotional support; these professionals are also expected to play an active role as part of a team to provide support to patients when making complex and difficult decisions. Therefore, in this study on effective team support, it was assumed that communication, leadership, and management skills were already acquired, and only those elements necessary for team SDM were adopted. The study results indicated that the present model of the Group’s SDM measure had a high goodness of fit, even without the questions regarding communication, leadership, and management skills. We believe that this is because we used data from workshops attended by many professionals who already have extensive clinical experience as specialists and are already using these skills on a daily basis. The data used in this study were obtained from two consecutive workshops. The first workshop was SDM skills training to support decision-making with relatively little conflict between patients and professionals. The second workshop was a team SDM program to support the complex and difficult decision-making process. Due to the influence of this training program, it is believed that a model can be completed with a high level of conformity, even if it lacks some items such as communication, leadership, and managerial skills.

### 4.2. Limitations of the Study

We did not grasp any prior knowledge on the participants, except for specialized areas such as palliative care. If we could grasp such knowledge in advance, the findings would be more clearly discussed. This study used the data of professionals who voluntarily participated in training workshops to learn about ACP. It is possible that compared with general professionals, the participants’ high motivation for decision support skewed the results. In addition, the fact that most participants were professionals with at least 25 years of clinical experience and extensive managerial experience indicates the possibility of bias.

Moreover, we only used training data targeting professionals, and we did not include any data of patients or their family. Therefore, in the future, it will be necessary to validate this Group’s SDM measure with several members, including patients, their families, and professionals with little experience in clinical decision support.

## 5. Conclusions

In this study, we validated the novel evaluation measure (Group’s SDM) for interprofessional SDM, with a team comprising multidisciplinary professionals, a patient, and the patient’s family, in order to support more complex and difficult decision-making. We could confirm its reliability (internal consistency) and validity, which showed the validation for its use in a practical and a training setting.

## Figures and Tables

**Table 1:** Specializations of participants who completed the second workshop (n = 171).

Specialization	Number of People	Percentage (%)
Nurse	86	50
Physician	28	16
MSW	18	11
Care Manager	14	8
Therapist	8	5
Counselor	4	2
Public Health Nurse	4	2
Pharmacist	3	2
Care Worker	2	1
Registered Dietitian	1	1
Other	3	2
Total	171	100

**Table 2:** Length of experience of participants who completed the second workshop (n = 171).

Years of Clinical Experience as a Specialist	Number of People	Percentage (%)
<5 years	20	12
≥5 to <10 years	20	12
≥10 to <15 years	24	14
≥15 to <20 years	33	19
≥20 to <25 years	37	21
≥25 years	37	22
Total	171	100

**Table 3:** Descriptive statistics for the 10 items of the Group’s SDM measure questionnaire.

	Median	Mean	SD	Minimum	Maximum	Corrected Item’s Total Correlation Coefficient
Group’s SDM 1	8.00	6.96	2.404	0	10	0.809
Group’s SDM 2	8.00	7.38	2.163	2	10	0.77
Group’s SDM 3	8.00	7.97	1.994	2	10	0.773
Group’s SDM 4	8.00	7.85	2.306	0	10	0.742
Group’s SDM 5	8.00	7.15	2.389	0	10	0.813
Group’s SDM 6	8.00	7.49	2.213	0	10	0.801
Group’s SDM 7	6.00	6.44	2.366	0	10	0.605
Group’s SDM 8	8.00	7.22	2.164	0	10	0.842
Group’s SDM 9	8.00	7.99	2.028	0	10	0.861
Group’s SDM 10	8.00	7.50	2.292	0	10	0.827

**Table 4:** Exploratory factor analysis for the Group’s SDM.

	Factor Loadings	Communality
Group’s SDM 1	0.83	0.69
Group’s SDM 2	0.79	0.66
Group’s SDM 3	0.80	0.67
Group’s SDM 4	0.77	0.61
Group’s SDM 5	0.84	0.68
Group’s SDM 6	0.82	0.69
Group’s SDM 7	0.62	0.48
Group’s SDM 8	0.86	0.79
Group’s SDM 9	0.89	0.82
Group’s SDM 10	0.85	0.72
Eigenvalue	6.90	
% of Total variance	68.94	

## Data Availability

The data used to support the findings of this study are available from the corresponding author upon request.
